# The Effect of Individual Factors on Health Behaviors Among College Students: The Mediating Effects of eHealth Literacy

**DOI:** 10.2196/jmir.3542

**Published:** 2014-12-12

**Authors:** WanChen Hsu, ChiaHsun Chiang, ShuChing Yang

**Affiliations:** ^1^Center for Teaching & Learning DevelopmentNational Kaohsiung University of Applied SciencesKaohsiungTaiwan; ^2^Graduate Institute of EducationNational Sun Yat-sen UniversityKaohsiungTaiwan

**Keywords:** demographic, health behavior, mediation, eHealth literacy, quantitative evaluation

## Abstract

**Background:**

College students’ health behavior is a topic that deserves attention. Individual factors and eHealth literacy may affect an individual’s health behaviors. The integrative model of eHealth use (IMeHU) provides a parsimonious account of the connections among the digital divide, health care disparities, and the unequal distribution and use of communication technologies. However, few studies have explored the associations among individual factors, eHealth literacy, and health behaviors, and IMeHU has not been empirically investigated.

**Objective:**

This study examines the associations among individual factors, eHealth literacy, and health behaviors using IMeHU.

**Methods:**

The Health Behavior Scale is a 12-item instrument developed to measure college students’ eating, exercise, and sleep behaviors. The eHealth Literacy Scale is a 12-item instrument designed to measure college students’ functional, interactive, and critical eHealth literacy. A nationally representative sample of 525 valid college students in Taiwan was surveyed. A questionnaire was administered to collect background information about participants’ health status, degree of health concern, major, and the frequency with which they engaged in health-related discussions. This study used Amos 6.0 to conduct a confirmatory factor analysis to identify the best measurement models for the eHealth Literacy Scale and the Health Behavior Scale. We then conducted a multiple regression analysis to examine the associations among individual factors, eHealth literacy, and health behaviors. Additionally, causal steps approach was used to explore indirect (mediating) effects and Sobel tests were used to test the significance of the mediating effects.

**Results:**

The study found that perceptions of better health status (t_520_=2.14-6.12, *P*<.001-.03) and greater concern for health (t_520_=2.58-6.95, *P*<.001-.003) influenced college students’ development of 3 dimensions of eHealth literacy and adoption of healthy eating, exercise, and sleep behaviors. Moreover, eHealth literacy played an intermediary role in the association between individual factors and health behaviors (Sobel test=2.09-2.72, *P*<.001-.03). Specifically, higher levels of critical eHealth literacy promoted students’ health status and their practice of multiple positive health behaviors, including eating, exercise, and sleep behaviors.

**Conclusions:**

Because this study showed that eHealth literacy mediates the association between individual factors and health behaviors, schools should aim to enhance students’ eHealth literacy and promote their health behaviors to help them achieve high levels of critical eHealth literacy. Although some of the study’s hypotheses were not supported in this study, the factors that influence health behaviors are complex and interdependent. Therefore, a follow-up study should be conducted to further explore how these factors influence one another.

## Introduction

### Background

The health behavior of college students is a topic that is worth exploring. According to Taiwan’s Health Promotion Administration of the Ministry of Health and Welfare, National Health Survey statistics showed that the percentage of college students aged 18-24 years who exercised regularly was 60.8% in 2002, 60.6% in 2005, and 55.1% in 2009. Furthermore, the percentage of students who ate breakfast daily was 65.8% in 2002, 62.6% in 2005, and 57.3% in 2009 [1]. Data from a 2010 survey showed a 2-hour difference between the number of hours students spent sleeping on school days (6.4 hours) and weekends (8.5 hours). These results indicate that Taiwanese college students’ exercise, eating, and sleep behaviors must be improved [2]. Many of the lifelong habits that jeopardize health are formed during childhood and adolescence [3]. As college students transition from adolescence into adulthood, their health habits may affect their future well-being. During this transition, those who have poor health habits may adopt better habits if they are given sound advice. Therefore, it is necessary and important to examine college students’ health behaviors because these behaviors affect students’ physical health and lifestyles in adulthood [4]. Health behavior is “any activity undertaken by a person who believes himself to be healthy, for the purpose of preventing disease or detecting it in an asymptomatic stage” [5]. A number of studies on health behavior have focused on eating and exercise [4,6]. Given that sleep is a basic physiological need and an essential element in maintaining physical and mental health, we will examine the health behaviors of college students by measuring their eating, exercise, and sleep behaviors.

Individual factors and eHealth literacy may affect one’s health behaviors. According to social cognitive theory [7], each factor possesses a self-regulating system that affects motivation and learner differentiation. Human behavior is influenced and affected by the individual, the behaviors of others, and the environment. This self-regulating system represents a process that is affected by bidirectional and interdependent associations between and among behaviors, environments, and personal experiences. Studies have found that certain factors, such as one’s health status, concern for health, and eHealth literacy, may shape an individual’s health behaviors [8].

eHealth literacy may mediate the association between demographic factors and health behaviors. The integrative model of eHealth use (IMeHU) suggests that the underlying social structure affects an individual’s health status, computer literacy, intrinsic interest in health, and perceived ability to use the Internet for health purposes [9]. The model also proposes macrolevel disparities in the social structures that are connected to health disparities through the microlevel conduits of eHealth literacy, motivation, and ability. That is, individuals with low eHealth literacy have less incentive to use the Internet to access health information and consider themselves to be incapable of using Internet-based health information. The IMeHU provides a parsimonious account of the connections among the digital divide, health care disparities, and the unequal distribution and use of communication technologies. However, few studies have examined the associations among individual factors, eHealth literacy, and health behaviors. Similarly, there is a lack of empirical evidence regarding the IMeHU. This study uses the IMeHU as a framework for examining the associations among individual factors, eHealth literacy, and health behaviors and to further validate the mediating effects of eHealth literacy on health behaviors and individual factors.

### Literature Review

#### Individual Factors Affect Health Behaviors

According to studies of college students in Taiwan, different individual factors affect people’s health behaviors [10,11]. These studies found that college students with better self-perceived health status or those enrolled in medical-related fields engaged in healthier behaviors and had more positive health-related attitudes and habits than other students had. For example, medical school students adhere to a more positive, health-promoting lifestyle than do not medical school students [10]. Furthermore, elders who rate their social interactive networks positively demonstrate better health and engage in exercise more consistently [12]. Accordingly, we propose hypothesis H1: based on certain individual factors, the health behaviors of college students can be predicted with relative accuracy.

#### eHealth Literacy Affects Health Behaviors

In recent years, studies of eHealth literacy have become more prevalent. Some studies focus on defining eHealth literacy [13-15], some design eHealth literacy programs [16,17], and others examine the effect and consequences of eHealth literacy [9]. People with high eHealth literacy are not only more inclined to use the Internet to find answers to health-related questions, but are able to understand the information that they find, evaluate the veracity of the information, discern the quality of different health websites, and use quality information to make informed decisions about health [9]. eHealth literacy affects an individual’s health information-seeking behaviors, including the initiative to search for and passively receive messages and then adopt health behaviors based on those messages, which ultimately affects one’s health outcomes [18]. Furthermore, those who possess higher levels of eHealth literacy may make healthier decisions, which in turn improve their health outcomes. Researchers have found that the use of health information on the Internet affects personal exercise habits, eating/food consumption habits, and activity habits [19], and that individuals with high eHealth literacy are more likely to permit evaluative procedures for colorectal cancer—a finding that further suggests that those who have better eHealth literacy may adopt more positive health behaviors [20]. Accordingly, we propose hypothesis H2: college students who possess better eHealth literacy engage in more positive health behaviors.

#### Individual Factors Affect eHealth Literacy

As one’s perception of his or her health status improves, his or her health literacy and health knowledge improves [21]. However, some studies show that health status does not affect one’s eHealth literacy [17]. Thus, it appears that the effect of health status on one’s ability to understand, use, or evaluate health information is inconclusive and requires further study. Studies on the impact of the frequency of health-related discussions on eHealth literacy show that discussions with parents or peers can promote eHealth literacy [17]. The 2003 US data on the health literacy of American adults found that individuals who often communicate with friends and family and who seek advice from professionals demonstrated higher levels of health literacy [22].

Few studies have examined the effect of students’ majors on their eHealth literacy. However, those who are oral health or dental majors tend to have better perceptions of their oral health behaviors than those who are not oral health or dental majors [11]. Similarly, medical school students may have a better cognitive understanding and perception of health information than nonmedical majors. Based on this information, we propose hypothesis H3: among college students, various individual factors (eg, health status, major, degree of health concern, and the frequency with which they engage in health-related discussions) can predict eHealth literacy.

#### eHealth Literacy Mediates the Association Between Individual Factors and Health Behaviors

Although studies have shown a connection among individual factors, eHealth literacy and health behaviors, no studies have examined the mediating effects. Because eHealth literacy plays an important role in individuals’ lives and health behaviors [9], the mediating effects are worthy of further exploration. According to Taiwanese college students, it is difficult for students to evaluate and adopt the suggested behaviors and activities [23]. Even after students critically evaluate the reliability of health information, they often adopt the behavior or activity that has the smallest effect on their health. Thus, hypothesis H4 proposes that eHealth literacy mediates the association between individual factors and health behaviors.

**Figure 1:**
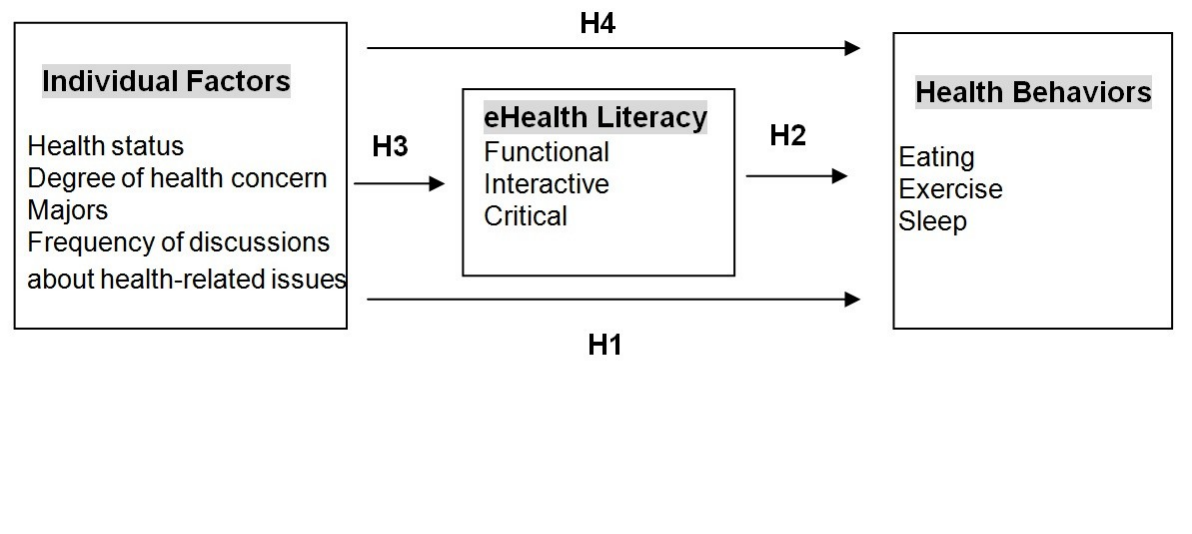
Theoretical framework of the study.

The 4 hypotheses that were developed based on the preceding discussions are shown in Figure 1.

## Methods

### Participants

According to the Ministry of Education and Statistics Department in Taiwan [24], there were a total of 1,037,632 college students in 2012. Accordingly, a sample of 350 was suggested for this investigation [25]. We determined that 500 students were needed for the official sample and estimated an effective questionnaire return rate of 80%. To make the sample more representative, the stratified cluster sampling method was employed. Using the region on a tiered basis and the class as the sampling unit, we extracted the desired projected number for each region sample based on the proportion of university students in the northern (n=292), central (n=146), southern (n=176), and eastern regions (n=11) of Taiwan. From March to May 2013, we surveyed a nationally representative sample of college students. A total of 625 questionnaires were distributed, including 64 unreturned and 36 incomplete questionnaires. Thus, 525 usable questionnaires were collected, resulting in an effective response rate of 84%. The sample’s sociodemographic and health information is displayed in Table 1.

**Table 1:** Sociodemographic and health information of the sample (N=525).

Variable and group		n (%)
**Health status**		
	Poor (score <4)	21 (4.0)
	General (score 5-7)	366 (69.7)
	Good (score >8)	138 (26.3)
**Degree of health concern**		
	Extremely unimportant	1 (0.2)
	Unimportant	13 (2.5)
	Average	215 (40.9)
	Important	240 (45.7)
	Extremely important	56 (10.7)
**Major**		
	Major in medical field	55 (10.5)
	Major in nonmedical field	470 (89.5)
**Frequency of discussion about health-related issues**		
	Never	0 (0.0)
	Seldom	118 (22.5)
	Sometimes	320 (60.9)
	Often	84 (16.0)
	Always	3 (0.6)

### Instrument

#### eHealth Literacy Scale

The eHealth Literacy Scale (eHLS) measures a student’s ability to seek, find, understand, and evaluate health information from electronic sources and apply this knowledge to address or solve a health problem. The 12-item eHLS, which was developed following a thorough review of the literature [14,26,27], includes the following 3 dimensions: functional, interactive, and critical eHealth literacy. Each dimension is evaluated using 4 items. The functional eHealth literacy dimension evaluates basic reading and writing skills and basic knowledge of health conditions and health systems. Interactive eHealth literacy refers to communicative and social skills that can be used to extract information and derive meaning from different forms of communication and to apply new information to changing circumstances. Critical eHealth literacy refers to advanced cognitive and social skills that can be applied to critically analyze information and to use this information to exert greater control over life events and situations that are related to individual and community-level goals.

An Amos 6.0 confirmatory factor analysis was used to examine the best measurement model. An analysis was conducted with Amos using maximum likelihood estimation. The participants were asked to accurately rate each eHLS item based on their own practices or beliefs using a 5-point Likert scale (1=strongly disagree, 5=strongly agree). We found that the current data adequately fit the eHLS model, which was divided into the following 3 dimensions (12 items total): functional eHealth literacy, interactive eHealth literacy, and critical eHealth literacy. With the use of Amos for the confirmatory factor analysis, a review of the fit indexes revealed a chi-square/df value of 3.02, a goodness of fit index (GFI) value of 0.95, an adjusted GFI (AGFI) value of 0.93, a comparative fit index (CFI) value of 0.95, and a root mean square error of approximation (RMSEA) value of 0.06. Furthermore, the chi-square test was significant (χ^2^_51_=153.8, *P*<.001).

#### Health Behavior Scale

The Health Behavior Scale (HBS) was also designed based on the literature [6,28-32] and the reference standard was based on Taiwan Ministry of Healthy Welfare [33] recommendations for individual eating, exercise, and sleep habits. The 3 health behavior dimensions included eating (eg, low-fat dairy foods and low-sugar cereals, vegetable and fruit consumption [more than 5 servings per day]), exercise (eg, exercise at least 3 times per week, monitor pulse while exercising), and sleep behaviors (eg, always have quality sleep, do not fall asleep during the day). Participants responded to the survey questions on a 5-point Likert scale (1=never, 5=always). High scores in the respective dimensions indicated more balanced eating habits, good and regular exercise habits, and good sleep habits and quality.

We found that the current data had a good fit with the model, which was divided into 3 dimensions (12 total items). With the use of Amos to conduct confirmatory factor analysis, a review of the fit indexes revealed a chi-square/df value of 2.74, a GFI value of 0.96, an AGFI value of 0.94, a CFI value of 0.96, and an RMSEA value of 0.06. Furthermore, the chi-square test was significant (χ^2^_50_=136.8, *P*<.001). However, a statistically nonsignificant overall chi-square value indicated good fit [34]; these standards reject many models with good fit and suggest other indicators [35]. In this study, the model showed an adequate fit to the data. We considered the 2 models to represent the best-fitting models for the eHLS and HBS structures.

### Background Information

Finally, we gathered the respondents’ background information, including information about their health status, their major, their degree of health concern, and the frequency with which they engaged in health-related discussions.

The students’ health status level (perceived psychological and physical status) was measured by asking them how well they currently felt with respect to their own psychological and physical condition on a scale from 1 (strongly unwell) to 10 (strongly well).

The students’ degree of health concern was measured by asking them about their health concerns and willingness to take appropriate action. The degree of importance of participants’ health was rated on a scale from 1 (extremely unimportant) to 5 (extremely important). A higher score indicated a higher level of concern regarding one’s health.

The major dimension was divided based on whether the participants were majoring in medical fields. In a subsequent analysis, the 2 groups were then transformed into dummy variables. We used the nonmedical group as the reference group.

The frequency with which students engaged in health-related discussions was measured based on participants’ responses on a scale from 1 (seldom) to 5 (always).

### Data Analysis

This study used Amos 6.0 to perform a confirmatory factor analysis to identify the best measurement models for the eHLS and HBS. We then used multiple regression analysis to examine the associations among individual factors, eHealth literacy, and health behaviors. Additionally, a causal steps approach was used to explore the indirect (mediating) effects [36] and Sobel tests [37] were used to test the significance of the mediating effects.

## Results

### Multiple Regression Analysis of Individual Factors Predicting eHealth Literacy

Table 2 indicates that nearly all the individual factors positively predicted the 3 dimensions of eHealth literacy, yielding low predictive explanatory powers of 7%-8%. Notably, all the individual factors emerged as significant indicators of the 3 dimensions of eHealth literacy, with the exception of the frequency of health-related discussions.

**Table 2:** Multiple regression analysis of individual factors predicting eHealth literacy.

Variable	Functional							Interactive							Critical						
	*R*	Δ*R* ^2^	*F*_4,520_	B	β	*t*_520_	*P*	*R*	Δ*R* ^2^	*F*_4,520_	B	β	*t*_520_	*P*	*R*	Δ*R* ^2^	*F*_4,520_	B	β	*t*_520_	*P*
Model summary	.29	.08	11.82					.29	0.8	11.55					.28	.07	10.87				
Health status				.14	.10	2.14	.03				.19	.11	2.34	.02				.28	.11	2.43	.02
Health concern				.37	.14	3.02	.003				.49	.15	3.24	.001				.56	.12	2.58	.01
Majors				.89	.15	3.45	.001				.73	.10	2.31	.02				.92	.09	2.04	.04
Frequency of discussions about health-related issues				.23	.08	1.79	.08				.34	.10	2.16	.03				.64	.13	2.81	.005

### Multiple Regression Analysis of Individual Factors and eHealth Literacy Predicting Health Behaviors

Two multiple regression analyses were performed to examine how well individual factors and health literacy predicted health behaviors. Table 3 reveals that the individual factors positively predicted the 3 health behavior dimensions, with a moderate level of predictive explanatory power for eating behaviors (23%) and a low level of predictive explanatory power for exercise (13%) and sleep behaviors (13%).

Table 3 further reveals that both health status and health concern positively predicted the 3 health behavior dimensions; major and the frequency of health-related discussions only demonstrated positive predictive power for eating behaviors. In addition, functional and critical eHealth literacy displayed positive predictive power for eating and exercise behaviors, whereas critical eHealth literacy only positively predicted sleep behaviors.

**Table 3:** Multiple regression analysis of individual factors and eHealth literacy predicting health behaviors.

Variable		Eating							Exercise							Sleep						
		*R*	Δ*R* ^2^	*F* (*df*)	B	β	*t* (*df*)	*P*	*R*	Δ*R* ^2^	*F* (*df*)	B	β	*t* (*df*)	*P*	*R*	Δ*R* ^2^	*F* (*df*)	B	β	*t* (*df*)	*P*
**Individual factors**																						
	Model summary	.48	.23	39.37 (4,520)					.37	.13	20.22 (4,520)					.37	.13	20.26 (4,520)				
	Health status				.44	.23	5.50 (520)	<.001				.64	.24	5.54 (520)	<.001				.59	.27	6.12 (520)	<.001
	Health concern				1.04	.30	6.95 (520)	<.001				.79	.17	3.65 (520)	<.001				.62	.16	3.42 (520)	.001
	Majors				.64	.08	2.02 (520)	.04				.71	.06	1.57 (520)	.12				.24	.03	0.64 (520)	.52
	Frequency of discussions about health-related issues				.37	.10	3.35 (520)	.02				.30	.06	1.33 (520)	.18				.23	.05	1.21 (520)	.23
**eHealth literacy**																						
	Model summary	.30	.08	17.05 (3,521)					.31	.09	18.03 (3,521)					.26	.06	12.90 (3,521)				
	Functional				.21	.16	3.48 (521)	.001				.27	.15	3.23 (521)	.001				.10	.07	1.44 (521)	.15
	Interactive				.09	.08	1.64 (521)	.10				.07	.05	0.99 (521)	.32				.07	.06	1.17 (521)	.24
	Critical				.11	.14	2.95 (521)	.003				.20	.19	3.98 (521)	<.001				.17	.20	4.03 (521)	<.001

### The Mediating Effects of eHealth Literacy on the Association Between Individual Factors and Health Behaviors

Table 4 is the result of a regression analysis of the individual factors and eHealth literacy as predictors of health behaviors. When comparing Tables 2 and 3, the standardized regression coefficients of the independent variables either decreased or were insignificant. Furthermore, a Sobel test of the mediating effect indicated that 6 of 13 paths were significant.

The 2 standardized regression coefficients were multiplied to compute a mediating effect, as presented in Table 5. Critical eHealth literacy mediated the association between health status and eating, exercise and sleep behaviors, showing mediating effect values of .015, .021, and .022, respectively. Functional eHealth literacy mediated the association between health concern and eating and exercise behaviors, with mediating effect values of .022 and .021, respectively. Critical eHealth literacy mediated the association between health concern and eating behaviors, yielding a mediating effect value of .017.

According to Baron and Kenny’s approach to statistical mediation analysis [36], it was further determined that critical eHealth literacy mediated the association between health status and health behaviors (see Table 6) and that the mediating effect was .058 units (.015+.021+.022). Functional/critical eHealth literacy mediated the association between health concern and health behaviors, with a mediating effect of .060 units (.022+.021+.017). These results indicate that participants who had better health status and greater concern for their health tended to have better functional and critical health literacy and were, therefore, more inclined to engage in positive health behaviors, especially positive eating behaviors.

**Table 4:** Regression analysis of individual factors and eHealth literacy predicting health behaviors.

Variable		Eating							Exercise							Sleep						
		*R*	Δ*R* ^2^	*F*_7,517_	B	β	*t*_517_	*P*	*R*	Δ*R* ^2^	*F*_7,517_	B	β	*t*_517_	*P*	*R*	Δ*R* ^2^	*F*_7,517_	B	β	*t*_517_	*P*
Model summary		.50	.24	25.00					.42	.16	15.65					.41	.15	14.48				
**Individual factors**																						
	Health status				.40	.21	5.02	<.001				.57	.21	4.97	<.001				.54	.25	5.66	<.001
	Health concern				.95	.27	6.31	<.001				.62	13	2.90	.004				.52	.13	2.87	.004
	Majors				.45	.06	1.43	.15				.38	.04	0.86	.39				.07	.01	0.19	.85
	Frequency of discussions about health-related issues				.30	.08	1.87	.06				.15	.03	0.68	.49				.13	.03	0.68	.50
**eHealth literacy**																						
	Functional				.12	.09	2.14	.03				.19	.10	2.31	.02				.04	.02	0.51	.61
	Interactive				.03	.02	0.53	.60				.02	.01	0.25	.80				.03	.02	0.41	.68
	Critical				.06	.08	1.80	.07				.16	.15	3.23	.001				.13	.15	3.27	.001

**Table 5:** Sobel test of the mediating effects of eHealth literacy on the association between individual factors and health behaviors.

Pathway	Independent to dependent variable, β		Sobel test	*P*
	Table 1	Table 3		
Health status→functional eHealth literacy→eating	.23	.21	1.96	.05
Majors→functional eHealth literacy→eating	.08	.06	1.32	.19
Health concern→functional eHealth literacy→eating	.30	.27	2.72	<.001
Health status→critical eHealth literacy→eating	.23	.21	2.19	.02
Majors→critical eHealth literacy→eating	.08	.06	1.17	.24
Health concern→critical eHealth literacy→eating	.30	.27	2.39	.01
Frequency of health-related discussions→critical eHealth literacy→eating	.10	.08	1.56	.11
Health status→functional eHealth literacy→exercise	.24	.21	1.95	.05
Health concern→functional eHealth literacy→exercise	.17	.13	2.09	.03
Health status→critical eHealth literacy→exercise	.24	.21	2.18	.02
Health concern→critical eHealth literacy→exercise	.17	.13	1.93	.05
Health status→critical eHealth literacy→sleep	.27	.25	2.23	.02
Health concern→critical eHealth literacy→sleep	.16	.13	0.01	.99

**Table 6:** Estimation of the causal mediating effects.

Pathway	Estimation of mediated effects
Health status→critical eHealth literacy→eating	.015
Health status→critical eHealth literacy→exercise	.021
Health status→critical eHealth literacy→sleep	.022
Health concern→functional eHealth literacy→eating	.022
Health concern→functional eHealth literacy→eating	.021
Health concern→critical eHealth literacy→eating	.017

## Discussion

### Overview

This study found that the participants who had better self-perceptions of their health status and stronger concern for their health exhibited better eHealth literacy and had an increased likelihood of adopting healthy eating, exercise, and sleep behaviors. Moreover, eHealth literacy mediated the association between individual factors and health behaviors.

### Better Health Status and Greater Health Concern Adopt More Positive Health Behaviors

This study found that the participants who had better perceived health status and greater concern for their health tended to adopt more positive health behaviors. Similarly, college students who majored in medical fields and who engaged in more health-related discussions demonstrated better eating behaviors. Thus, hypothesis 1 was largely supported. Consistent with social cognitive theory [7], an individual’s health status and health concern are key factors that prompt him or her to adopt health behaviors [10,12]. It appears that college students who have better perceived health status and greater concern for their own health pay greater attention to their health and are more willing to engage in appropriate health behaviors.

### Medical Majors, Better Health Status, and Greater Health Concern Have Better eHealth Literacy Development

The study also revealed that the participants who majored in medical fields, had better perceived health status, and had greater concern for their own health tended to have better eHealth literacy than other students. Additionally, the participants who frequently engaged in health-related discussions had better critical eHealth literacy. Therefore, consistent with previous studies [22,23,38], hypothesis 3 was largely supported: college students who had better perceived health and paid more attention to their health were more likely to seek and evaluate health information.

The framework for health literacy identifies 3 major areas of potential intervention and forms the organizational principles of intervention [8]. The framework also illustrates that educational, health, and cultural and social factors may influence health literacy and may ultimately contribute to health outcomes and costs. Health literacy involves a range of social and individual factors, including cultural and conceptual knowledge, listening, speaking, writing, and reading skills. For example, a previous study found that performance on health literacy tasks was related to education, income, country of birth, age, and race/ethnicity [39]. Specifically, individuals with higher educational attainment and higher income demonstrate higher levels of health literacy [9]. For example, medical school students have higher incomes and more medical knowledge and, therefore, possess greater eHealth literacy than non-medical school students.

### No Prediction of Medical Majors and Health-Related Discussions for Exercise and Sleep Behaviors

However, this study also found that the participant’s major and the frequency with which he/she engaged in health-related discussions did not predict exercise and sleep behaviors. Because eHealth literacy relates to one’s context of relevant medical knowledge when assessing the quality of health information and when making decisions that promote one’s health [9], students who major in medical fields are likely to have greater knowledge of medicine. Therefore, medical school students have better health literacy than non-medical school students. The knowledge, attitude, and practice (KAP) model advocates that once an individual receives relevant information, the individual will develop the expected responses, thus triggering behavior that is consistent with one’s attitude [40]. However, outcomes and efficacy are not necessarily aligned [7]. In other words, what one knows does not necessarily equate to what one does.

Similarly, the concept of the “KAP gap” states that even if an individual is introduced to new ideas or practices and has a positive attitude toward these new ideas or practices, the individual will not necessarily adopt the behavior [41]. This disconnect results in inconsistency and creates a sense of cognitive dissonance. This problem is especially apparent with respect to new preventive ideas or practices because they often lack positive benefits and advantages. Thus, the effect is more ambiguous and individuals are more prone to experiencing a KAP gap. This tendency may explain why medical majors and those who frequently engage in health discussions do not necessarily implement their knowledge into actions. Future studies are needed to examine the motivation for one’s decision to adopt, reject, or discontinue a healthy behavior (ie, what leads to the KAP gap).

### Less Influential of Functional and Interactive than Critical eHealth Literacy

Partially consistent with IMeHU [9], we found that critical health literacy played a key role in health status and health behaviors. Functional health literacy positively predicted eating and exercise behaviors, and critical health literacy positively predicted the 3 dimensions of health behaviors. However, interactive health literacy did not contribute to the 3 dimensions of health behavior. Accordingly, hypothesis 2 was partially supported. Individual health information literacy strengthens one’s intent to search for and apply health information and influences one’s health decision making and engagement in healthy behaviors [18]. Thus, the current study concludes that both functional and critical health literacy and dialog contribute to the adoption of positive health behaviors, especially eating and exercising behaviors.

However, functional eHealth literacy was not found to affect sleep behaviors, and interactive eHealth literacy was not found to affect any of the health behavior dimensions. Therefore, it is inferred that the factors that influence health behaviors are complex. The promotion of individual eHealth literacy affects the retention of eHealth information and subsequently influences future actions [9]. According to involvement theory [42], critical eHealth literacy may motivate individuals to seek and evaluate the quality of health information. In other words, individuals may attempt to gain as much information as possible and then evaluate and use the information to reach an optimal decision. The processing involved in functional and interactive eHealth literacy does not engage as deeply with an issue as that of critical eHealth literacy. Thus, functional and interactive eHealth literacy are less influential than critical eHealth literacy.

### Intermediary Role of eHealth Literacy in the Association Between Individual Factors and Health Behaviors

The findings also revealed that functional and critical eHealth literacy mediated the association between individual factors and health behaviors. Thus, hypothesis 4 was partially supported. We found that functional eHealth literacy only mediated the association between health concern and 2 health behavior dimensions (eating and exercise behaviors). Critical eHealth literacy mediated not only the association between health concern and eating behaviors, but also the association between health status and all 3 health behavior dimensions. Therefore, college students’ critical eHealth literacy influenced the association between perceived health status and the 3 health behavior dimensions. These findings are consistent with the belief that critical literacy is crucial to assess the quality of health information because laypersons risk harming themselves in their self-diagnosis and treatment when they lack the required background knowledge to correctly interpret information [43].

### Conclusions

This study used the IMeHU to explore the associations among individual factors, eHealth literacy, and health behaviors. We hope that the findings will stimulate further debate about how a health education framework can be translated into practical approaches and will contribute to further refinement of the eHealth literacy concept. The study showed that eHealth literacy played an intermediary role in the association between individual factors and health behaviors. Thus, we suggest that schools strengthen college students’ eHealth literacy and promote positive health behaviors based on the current level of eHealth literacy among students [44]. Schools can further use the 6 core skills of eHealth literacy [45] (eg, traditional literacy, health literacy, information literacy, scientific literacy, media literacy, and computer literacy) to develop healthy eating, exercising, and sleeping behavior guidelines and to incorporate these guidelines into health education programs. Moreover, the current findings demonstrated that critical health literacy is a key competence in promoting individual health behaviors. Thus, it is suggested that the development of critical eHealth literacy and the promotion of positive health behaviors among college students require further investigation.

As this study indicated, the IMeHU [9] provides a parsimonious framework that is currently lacking in the extant literature. The examination of the potential effect of eHealth literacy on health behavior presents a unique challenge because it involves a complex interplay of basic literacy skills, the ability to successfully navigate the dominant language framework (English) and culture that is utilized for Web-mediated communication, and sufficient levels of technology adoption and proficiency [46]. Although some research hypotheses failed to gain support in this study, given that the factors that influence health behaviors are complex and interdependent, a future study should be conducted to explore how these factors influence one another. Further research is also needed to refine and verify the parsimonious framework of the IMeHU [9].

## Abbreviations

CFI: comparative fit index
GFI: goodness of fit index
HBS: Health Behavior Scale
IMeHU: integrative model of eHealth use
KAP: knowledge, attitude, and practice
RMSEA: root mean square error of approximation
